# Effect of Co-Solvents, Modified Starch and Physical Parameters on the Solubility and Release Rate of Cryptotanshinone from Alcohologels

**DOI:** 10.3390/molecules29245877

**Published:** 2024-12-12

**Authors:** Justyna Kobryń, Patryk Demski, Bartosz Raszewski, Tomasz Zięba, Witold Musiał

**Affiliations:** 1 Department of Physical Chemistry and Biophysics, Faculty of Pharmacy, Wroclaw Medical University, Borowska 211A, 50-556 Wroclaw, Poland; justyna.kobryn@umw.edu.pl (J.K.); patryk.demski97@gmail.com (P.D.); 2 Department of Food Storage and Technology, Faculty of Biotechnology and Food Science, Wroclaw University of Environmental and Life Sciences, Chełmońskiego 37, 51-630 Wroclaw, Poland; b.raszewski31@gmail.com (B.R.); tomasz.zieba@upwr.edu.pl (T.Z.)

**Keywords:** cryptotanshinone, alcohologel, release, potato starch, ATR-FTIR, PXRD, ESI-MS

## Abstract

(1) Background: The aim of the work was to investigate the influence of selected physico-chemical factors on the solubility and release rate of CT (cryptotanshinone) in alcohologels. (2) Methods: The alcohologels of methylcellulose (MC), hydroksyethylcellulose (HEC), polyacrylic acid (PA) and polyacrylic acid crosspolymer (PACP) with CT were prepared and/or doped with native potato starch (SN) and modified citrate starches (SM2.5 and SM10). The analytical methods included evaluation of CT release profiles, Fourier transform infrared spectroscopy (ATR-FTIR), differential scanning calorimetry (DSC), powder X-ray diffraction (PXRD) and electrospray ionization mass spectrometry (ESI-MS), and scanning electron microscope (SEM) images were performed. (3) Results: The release and decomposition kinetics of CT in relation to the phosphate buffer solution (PBS) and methanol were observed. The amount of cryptotanshinone (CT) released into PBS was significantly lower (2.5%) compared to its release into methanol, where 22.5% of the CT was released into the model medium. The addition of SM2.5 to the alcohologel significantly increased the CT content to 70% in the alcohologel preparation containing NaOH (40%), and this enhanced stability was maintained for up to two months. The ATR-FTIR exhibited interactions between PA and 2-amino-2-methyl-1,3-propanediol (AMPD) as well as between PA and NaOH in case of the alcohologels. Moreover, it indicated the interaction between CT and NaOH. PXRD diffractograms confirmed the FTIR study. (4) Conclusions: The study observed the influence of a number of factors on the solubility and release rate of CT, as: alkalizers and their concentration, SM2.5 addition. The transition of CT in the presence of NaOH to the tanshinone V sodium (T-V sodium) form was suspected.

## 1. Introduction

Cryptotanshinone (CT), also referred to as tanshinone C, is a natural component of *Salvia miltiorrhiza.* It belongs to a class characterized by a quinone diterpene structure [1]. While CT is insoluble in water, it exhibits good solubility in organic solvents such as chloroform, methanol or ethanol, among others [2,3]. Our research previously established that the solubility of CT increased in alkaline solvents when ethanol was used as a co-solvent [4]. We focused on selected alkaline substances that improved the solubility and stability of CT in aqueous–alcohol solution: 2-amino-2-methyl-1,3-propanediol (AMPD) and sodium hydroxide (NaOH). AMPD, an organic primary amine, is freely soluble in water and alcohols [5]. It is commonly used in cosmetic formulations, particularly in eye-area products at a concentration of 2% [6,7]. Ethanol has been used as solvent or co-solubilizer of organic ingredients including diterpenoid tanshinones [8,9,10,11]. CT stability is crucial for its activity. A decrease in the stability of CT can lead to a decrease in its activity. The decrease may be related to structural changes in the molecule, among other things.

Numerous studies have demonstrated that the chemical structures of organic compounds significantly influence antibacterial activity [12,13,14]. Danshen, a traditional medicine endemic to Chinese herbal practice, is derived from the root of *Salvia miltiorrhiza*. The preparation contains several active compounds that differ slightly in structure, many of which have been tested for antibacterial properties in recent decades. The most extensively studied molecules include cryptotanshinone (CT), dihydrotanshinone (DHT), tanshinone I (T-I) and tanshinone IIA (T-IIA) [11,15,16,17]. Both CT and DHT have been known to exhibit activity against Gram-positive bacteria, although they do not demonstrate activity against Gram-negative strains [18]. T-I is active against *Mycobacterium tuberculosis*, while T-IIA has been shown to reduce inflammation caused by *Helicobacter pylori* [19]. Additionally, the tanshinones have demonstrated antiviral activity [20]. T-IIA and its sulfonate derivative exhibit a range of biological activities. They are realized via redox signaling pathway [21,22,23,24].

Cryptotanshinone (CT) has garnered attention for its potential therapeutic properties, including anti-inflammatory, antioxidant and anticancer effects. Our study investigated formulation possibilities for potential topical application of CT. Several studies have explored its effects when applied topically, particularly concerning skin conditions and wound healing. M. Song et al. [25] have applied CT in sodium carboxymethyl cellulose to mice with diabetic wounds. N.-G. Kang et al. [26] have confirmed the treatment of *Acne vulgaris* with CT. They tested 0.1% CT as a solution in ethanol and hydrogenated castor oil and an emulsion with carbomer, triethanoloamine (TEA) and polysorbate 60, among others. Hydrogels are central to our research due to their essential applicative properties, including biocompatibility, stability across a wide pH range, ease of preparation and the ability to control the release of active substances. Additionally, these formulations can help to maintain moisture and create an optimal environment for tissue healing. They can enhance the delivery and stability of compounds like cryptotanshinone for topical applications. Z. Wang et al. [27] investigated a cryptotanshinone-loaded niosomal hydrogel, highlighting its acne-healing properties by demonstrating improved skin penetration and sustained release without skin irritation in mice model. They used the niosome structure to improve CT solubility and incorporated niosomal capsules into a polyacrylate hydrogel.

As a readily available, biodegradable, easily chemically modified and cost-effective material, starch is utilized in the agricultural industry. It serves as a carrier substance and as a preservative for chemical fertilizers, such as those containing nitrogen or phosphorus [28,29,30,31]. M. Salimi et al. [28] utilized starch in combination with acrylic acid and acrylamide polymers, along with charcoal, to control the release of urea. S. Lü et al. [32] achieved slow release of urea using starch acetate crosslinked with carboxymethyl xanthan gum. Additionally, K. Zhong et al. [33] identified sulfonated corn starch crosslinked with polyacrylic acid as a superadsorbent for phosphate rock. In our study, we used native potato starch and citric acid-modified starch as potential carriers, based on our previous research on the release of lidocaine hydrochloride from hydrogels incorporating starch. The implementation of the starch resulted in prolonged drug release [34]. Moreover, we have not observed any case studies on the use of starch as a CT carrier. So far, CT has been applied in the forms of ethosomes [35] and niosomes [27], as it increases a transdermal pathway for active pharmaceutical ingredients (APIs). In another study, the addition of dispersed silicon to a cataplasm with tanshinone IIA was intended to improve the release and skin-penetrating capabilities of the drug [36]. The application of starch is an innovative method and may be of high importance for drug release as it is in other cases of different kinds of starch [28,32].

FTIR and ESI-MS spectroscopic methods are widely used in the analysis of the structure of synthetic, natural substances and interactions between them or ingredients acting as carriers [37,38,39,40]. CT and tanshinone IIA structure, as well as interactions between tanshinone IIA and chitosan or withsynthetic polymers were tested using FTIR [37,41,42]. HPLC and MS analytical methods were used for quantitative analysis of CT, CT’s metabolite structures, tanshinone I and tanshinone IIA contents and danshen composition [43,44,45,46]. The aim of our research was to utilize hydrogels as carriers for cryptotanshinone dissolved in ethanol under alkaline conditions of AMPD or NaOH. We investigated the influence of various factors on the release profile of cryptotanshinone from the hydrogel, as well as the effects of the following factors: elevated temperature, alkali concentration and sonication treatment. The presence of native and modified starch on the stability of cryptotanshinone in evaluated hydrogel systems was also studied.

## 2. Results

### 2.1. Determination of pH

The pH of the alcohologel formulations with CT were in the range of 5.00 to 13.39, whereas the pH of the alcohologels without CT was in the range of 6.46 to 6.81. The composition of the alcohologel with CT, NaOH and polyacrylic acid crosspolymer (N-PACP) showed the most alkaline character. The formulation of the alcohologel with CT, NaOH, polyacrylic acid and modified starch with 10% citric acid (N-PA-SM10) exhibited the most acidic character (Table 1). Statistically significant differences (*p* < 0.05) occurred between the pH values of most formulations, except for alcohologel with CT, AMPD and polyacrylic acid (A-PA); alcohologel with CT, AMPD, polyacrylic acid and native starch (A-PA-SN); A-PA-SN and alcohologel with CT, AMPD and polyacrylic acid prepared with higher concentrations of alkalizers and ultrasonication at 60 °C (A-PA(*)); A-PA(*) and alcohologel with CT, NaOH and polyacrylic acid prepared in standard conditions (N-PA); A-PA-SN and alcohologel with CT, NaOH, polyacrylic acid and native starch (N-PA-SN); and alcohologel with CT, NaOH and methylcellulose (N-MC) and alcohologel with CT, NaOH and hydroxyethylcellulose (N-HEC). Further studies were performed on polyacrylic acid (PA)-based alcohologel formulations only, due to pH values, as is explained in Section 3.

### 2.2. CT Release Evaluation

**Figure 1:**
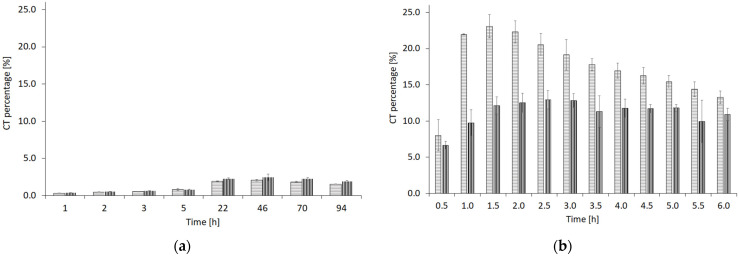
Release percentage of CT: (**a**) into PBS from alcohologels A-PA(i) (the bars with vertical lines) and N-PA(i) (the bars with horizontal lines) over 94 h, 37 ± 0.5 °C, (**b**) into methanol from alcohologels A-PA (the bars with vertical lines) and N-PA (the bars with horizontal lines) over 6 h, 22 ± 0.5 °C, *n* = 3. (i) refers to the alcohologels containing 20% ethanol.

The release profile of CT from alcohologels into methanol demonstrated that the amount of CT released was approximately ten times greater, over a release period that was more than 15 times shorter, in comparison to the release into phosphate buffer. Both the release of CT into the aqueous system and into methanol were characterized by distinct stages of increasing and decreasing concentration (Figure 1).

An attempt to determine compliance with the kinetic models concerned the stages of increasing and decreasing the amount of CT released. The table below shows the kinetic parameters and the values of the coefficient of determination r^2^ (Table 2). The statistical dependence of most shape constant β values obtained by the Weibull method was significant (*p* < 0.05), except for those of CT released into methanol from N-PA. There were no statistically significant differences (*p* > 0.05) between scale constant α values for most CT released into PBS (A-PA(i) and N-PA(i)).

### 2.3. Determination of CT Stability

**Figure 2:**
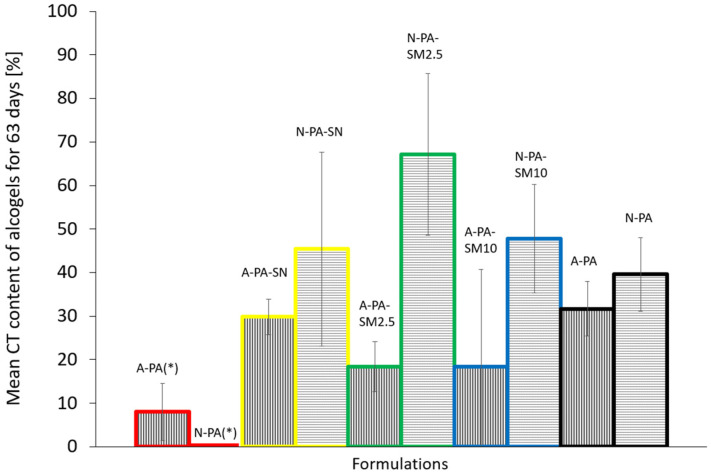
Mean content of CT in alcohologels stored in cool conditions for 60 days depending on preparation parameters: ultrasound and 60 °C (the bars with red lines), adding SN (the bars with yellow lines), SM2.5 (the bars with green lines) and SM10 (the bars with blue lines) with AMPD (A) and NaOH (N) as compared to referent alcohologels (A-PA and N-PA) (the bars with black lines), *n* = 5. The asterisk (*) indicates alcohologels preparation method with higher concentrations of alkalizers and ultrasonication at 60 °C.

The stability assay of CT in polyacrylic acid (PA) polymer-based alcohologels was examined. The effect of various preparation parameters, such as type of alkaline reagent, ultrasound at elevated temperature and addition of native and modified starch on the CT content after 60 days, were evaluated. Examination of mean CT content in alcohologels with modified parameters showed the disintegration of CT. In the samples subjected to ultrasound treatment at 60 °C for 10 min in 18% NaOH as well as in 2.3% AMPD solutions (A-PA(*), respectively), severe disintegration was exhibited. Complete disintegration was observed in N-PA(*). Slight CT degradation (about 30%) was observed in the presence of 0.36% NaOH solution and SM2.5 (N-PA-SM2.5) (Figure 2). An ANOVA test was applied separately to formulations with AMPD and NaOH. Statistically significant differences (*p* < 0.05) occurred between the CT content values of most formulations with AMPD, except for A-PA and A-PA-SN; A-PA and A-PA-SM10; the alcohologel with CT, NaOH, polyacrylic acid and modified starch with 2.5% citric acid (A-PA-SM2.5) and A-PA-SM10; and A-PA-SN and A-PA-SM10. Only one pair of the formulations with NaOH (N-PA and N-PA-SM2.5) showed statistically significant differences at a significance level of 0.05.

### 2.4. ATR-FTIR Analysis

**Figure 3:**
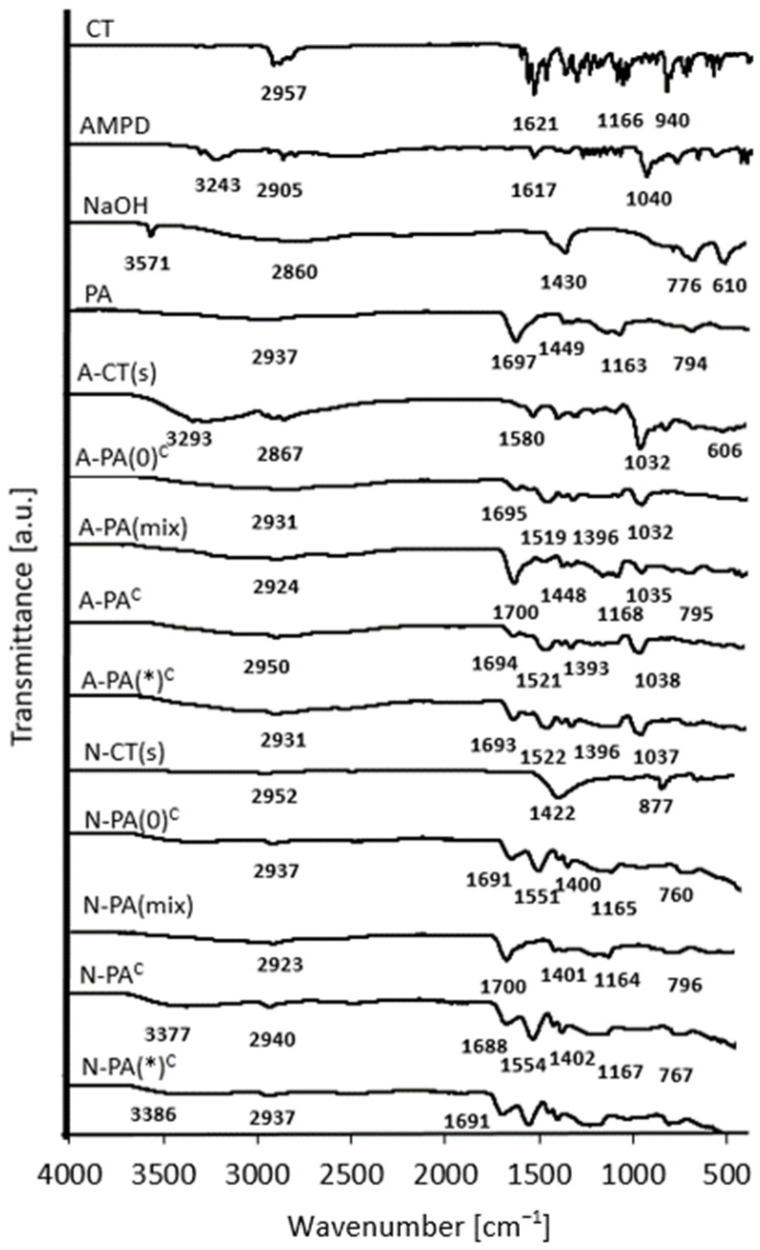
ATR-FTIR spectrogram of the raw substances, suspensions (s), cryo-alcohologels and physical mixtures (mix); c index indicates cryo-alcohologels. The asterisk (*) indicates alcohologels preparation method with higher concentrations of alkalizers and ultrasonication at 60 °C.

To evaluate interactions between the alcohologels and suspension components, and CT, as well as between PA and the solvents, infrared spectra were recorded. Comparison of the ATR-FTIR spectra of cryo-alcohologels containing PA and AMPD (without CT (A-PA(0)^c^), containing CT (A-PA^c^) and prepared with higher concentrations of alkalizers and ultrasonication at 60 °C (A-PA(*)^c^)) with the corresponding suspension (A-CT(s)) revealed a peak indicative of second-order amide group vibrations. This peak was observed at approximately 1520 cm^−1^ for the cryo-alcohologels and at 1580 cm^−1^ for A-CT(s) [47]. The peaks observed in the range of 3328 to 3183 cm^−1^ correspond to vibrations associated with both amine N-H and hydroxyl O-H groups in AMPD. In the spectrum of A-CT(s), a peak located at 3293 cm^−1^ may be attributed to N-H vibration from the amide group and O-H bonds [48]. The peaks observed around 1700 cm^−1^, corresponding to the stretching vibration of C=O [49,50], were evident in the cryo-alcohologel formulations as well as in the raw materials containing PA. Strong C-N peaks between 1040 and 1032 cm^−1^ were clearly visible in the spectra with AMPD. Characteristic absorption of O-H was identified at 687.5 cm^−1^ in AMPD, at 604.1 cm^−1^ in the physical mixture of AMPD and PA (A-PA(mix)) and at 606 cm^−1^ in the A-CT(s) spectra [51]. These peaks were only faintly observed in alcohologel formations (A-PA(0)^c^, A-PA^c^ and A-PA(*)^c^). The spectra of cryo-alcohologels containing PA and NaOH (designated as N-PA(0)^c^, N-PA^c^ and N-PA(*)^c^) exhibited a peak associated with the antisymmetric stretching of the carboxylate COO^¯^ group at approximately 1550 cm^−1^ [52], along with broad peaks around 3370 cm^−1^ corresponding to O-H hydroxy groups [53]. Notably, these features were absent from the spectra of the corresponding physical mixture (N-PA(mix)) and N-CT(s). Additionally, a sharp peak indicating the free O-H group was observed at 3571.5 cm^−1^ in the NaOH spectrum [54], which was not present in the other spectra. The standard CT spectrum revealed the presence of several groups, including C-H stretching bonds at 2956.8and 2925.5 cm^−1^, C=C or C=O bonds in the range of 1683.5 to 1620.9 cm^−1^, C-C bonds located within the aromatic ring (1620.9 to 1447.8 cm^−1^) and C-O bonds (1294.0 to 994.6 cm^−1^) [51,55]. Vibrations associated with aliphatic groups were observed across all spectra (Figure 3).

### 2.5. Powder X-Ray Diffraction (PXRD) Analysis

**Figure 4:**
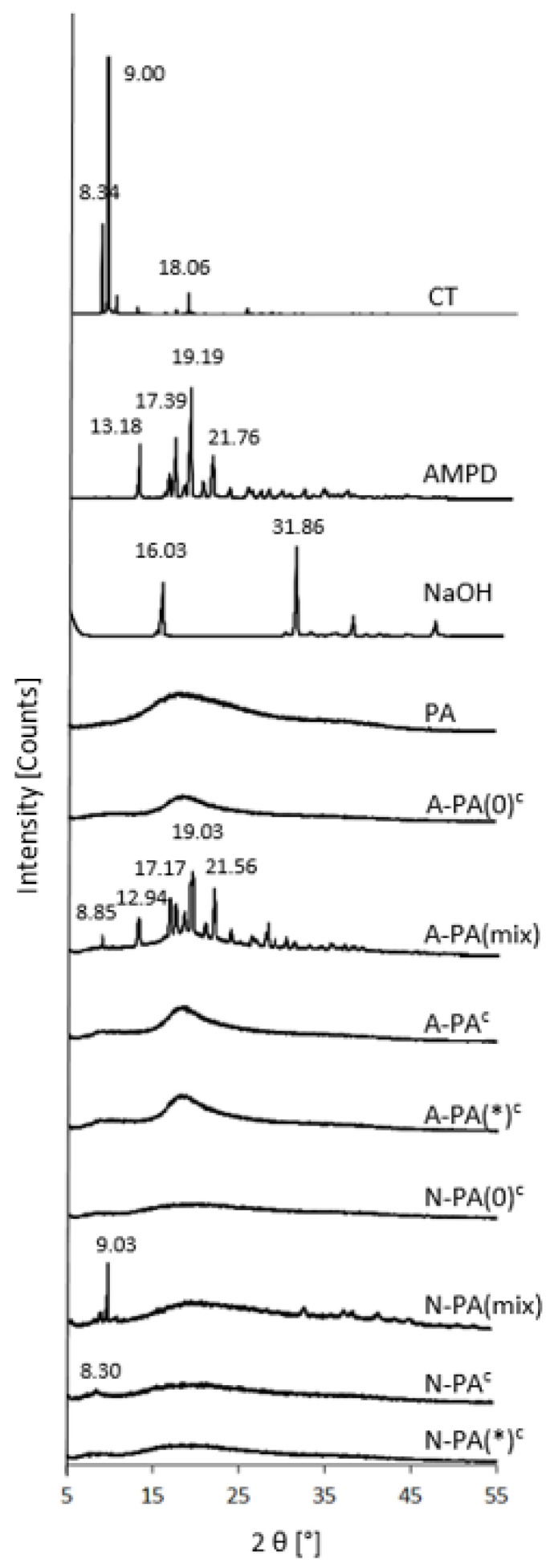
PXRD diffractograms of the raw substances, suspensions (s), cryo-alcohologels and physical mixtures (mix); c index indicates cryo-alcohologels. The asterisk (*) indicates alcohologels preparation method with higher concentrations of alkalizers and ultrasonication at 60 °C.

The diffractograms of CT-containing cryo-alcohologels (A-PA^c^, N-PA^c^) were compared to the diffractograms of their individual components (CT, AMPD, NaOH and PA), as well as to those of their mixture (A-PA(mix), N-PA(mix)) and cryo-alcohologels without CT (A-PA(0)^c^ and N-PA(0)^c^). The diffractogram of CT exhibited prominent diffraction peaks at 2θ values of 8.34, 9.00 and 18.06°. In the diffractogram of N-PA^c^, a reflection peak was observed at 2θ = 8.30°. Additionally, a shoulder peak appeared at 8.34° in the diffractograms of A-PA^c^ and A-PA(*)^c^ (Figure 4).

### 2.6. Differential Scanning Calorimetry (DSC)

**Figure 5:**
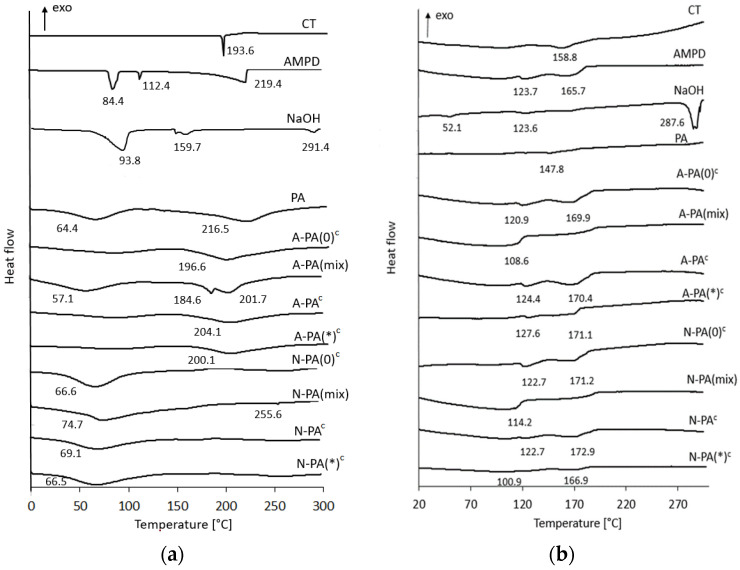
DSC thermograms of the raw substances, suspensions (s) alcohologels and physical mixtures (mix); c index indicates cryo-alcohologels at the first (**a**) and second (**b**) cycle of heating. The asterisk (*) indicates alcohologels preparation method with higher concentrations of alkalizers and ultrasonication at 60 °C.

DSC analysis showed thermograms of the pure substances (CT, AMPD, NaOH, PA), the cryo-alcohologels (A-PA(0)^c^, A-PA^c^, A-PA(*)^c^, N-PA(0)^c^, N-PA^c^, N-PA(*)^c^) and the physical mixtures of the components (A-PA(mix), N-PA(mix)). DSC thermograms of the first cycle of heating of CT, AMPD, NaOH and PA exhibited endothermic peaks (at 193.6 and 84.4, 112,4, 219.4 and 93.8, 159.7, 291.4 and 64.4, 216.5 °C, respectively) [41,56,57,58]. Comparison of the thermograms of the physical mixture A-PA(mix) with thermograms of the corresponding cryo-alcohologels (A-PA(0)^c^, A-PA^c^, A-PA(*)^c^) revealed the occurrence of a peak at 184.6 °C only in the case of the physical mixture. Similarly, a comparison of the thermograms of the physical mixture N-PA(mix) with those of the cryo-alcohologels revealed that only the thermogram of the mixture exhibited a peak at 255.6 °C (Figure 5a). The DSC thermograms of the second cycle of heating of CT, AMPD, NaOH and PA exhibited endothermic peaks (at 158.8 and 123.7, 165.7 and 52.1, 123.6, 287.6 and 147.8 °C, respectively) (Figure 5b).

### 2.7. ESI-MS Evaluation

**Figure 6:**
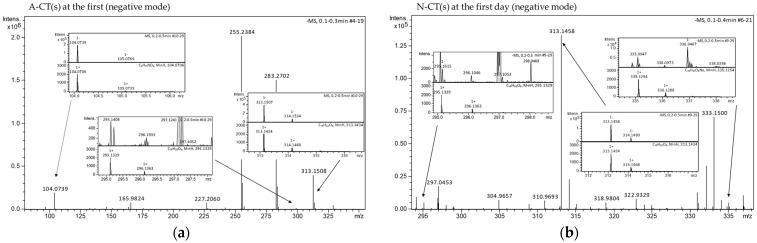
ESI-MS spectra of suspensions containing CT in negative mode prepared with 1% AMPD solution after the first day (**a**) and 0.36% NaOH solution after the first day (**b**).

**Figure 7:**
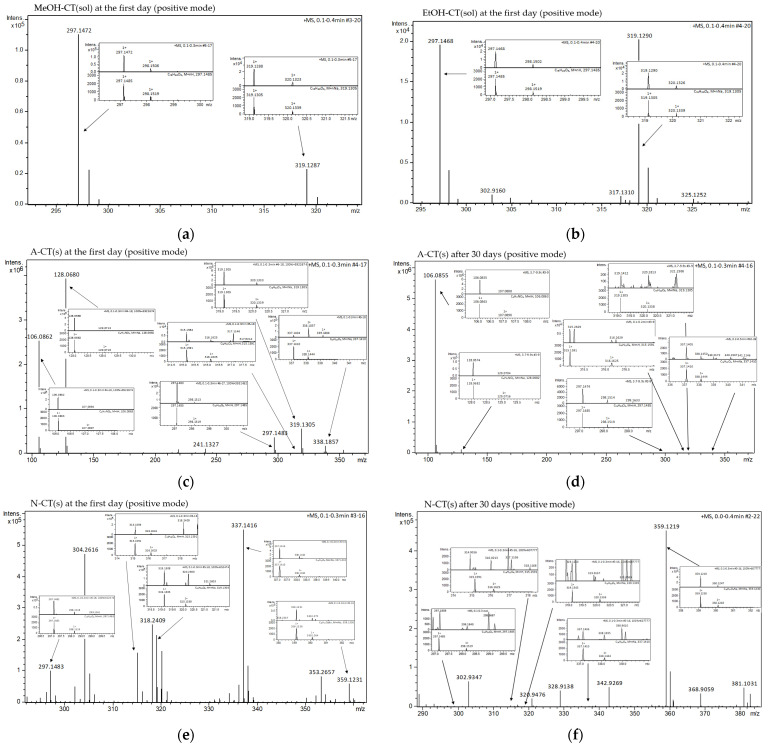
ESI-MS spectra of suspensions with CT prepared with: methanol (**a**), ethanol (**b**), 1% AMPD solution after the first day (**c**) and after 30 days (**d**), 0.36% NaOH solution after the first day (**e**) and after 30 days (**f**).

In the ESI-MS studies, the peaks with the *m*/*z* values corresponding to the molecular weight of CT, its derivatives and the solvents, which produced adduct ions, containing proton [M + H]^+^ or without proton [M − H]^−^ and/or containing sodium cation [M + Na]^+^, were recorded (Figure 6 and Figure 7). The ESI-MS spectra revealed standard CT peaks of adduct ions containing proton [M + H]^+^ at m/z 297.1485, without proton [M − H]^−^ at *m*/*z* 295.1329 and containing sodium cation [M + Na]^+^ at *m*/*z* 319.1305.

Comparing the *m*/*z* values and isotopic forms to the established standards, none of the tested formulations exhibited a satisfactory match to the peak corresponding to the CT in negative ionization mode (Figure 6). Therefore, the studies of ESI-MS in negative mode were not continued after 30 days. Concurrently, peaks of tanshinone V (T-V) [17,59] were found with negative ionization at *m*/*z* 313.1434 [M − H]^−^ in the formulations of CT containing AMPD and NaOH (Figure 6a,b).

Peaks matched to CT at a positive ionization were found in formulations from the first day from 1% AMPD and 0.36% NaOH solutions, at *m*/*z* 297.1483 and 297.1482, respectively (Figure 7c,e). After 30 days of storage, the peaks that matched the established CT standard values were noticed only in the 1% AMPD solution at *m*/*z* 297.1474 (Figure 7d). In assessing the fit, the distribution of isotope peaks was also considered. Overall, a decrease in the intensity of CT peaks was observed after 30 days of sample storage.

The base peaks of T-V [17,59] were identified at m/z 315.1591 [M + H]^+^, *m*/*z* 337.1410 [M + Na]^+^ and *m*/*z* 359.1230 [M − H + 2Na]^+^. These peaks were detected in the formulation of CT with 0.36% NaOH both on the first day and after 30 days of storage. A fluctuating intensity pattern was observed; specifically, the intensities of [M + H]^+^ and [M + Na]^+^ decreased while the intensity of [M − H + 2Na]^+^ increased (Figure 7e,f). In the formulation of CT containing AMPD, peaks at *m*/*z* 315.1591 [M + H]^+^ and *m*/*z* 337.1410 [M + Na]^+^ were present at the beginning of the experiment and after 30 days of storage (Figure 7e,f). The intensity of the first peak remained stable over time, whereas the intensity of the second peak decreased.

The following table summarizes the *m*/*z* values for the peaks of the spectra of the tested suspensions (Table 3).

### 2.8. SEM Images

**Figure 8:**
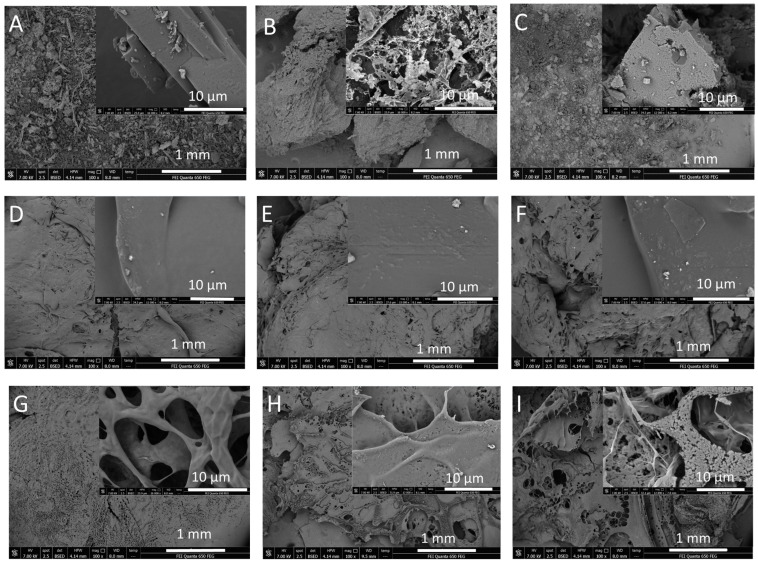
SEM micrographs of CT (**A**), the suspensions: A-CT(s) (**B**), N-CT(s) (**C**) and the cryo-alcohologels: A-PA(0)^c^ (**D**), A-PA^c^ (**E**), A-PA(*)^c^ (**F**), N-PA(0)^c^ (**G**), N-PA^c^ (**H**) and N-PA(*)^c^ (**I**), from 100 to 16,000 magnification.

SEM images of CT, vacuum-dried suspensions (A-CT(s), N-CT(s)) and cryo-alcohologels (A-PA(0)^c^, A-PA^c^, A-PA(*)^c^, N-PA(0)^c^, N-PA^c^ and N-PA(*)^c^) are presented (Figure 8).

## 3. Discussion

### 3.1. Determination of pH

pH determination was employed to adjust the alcohologels to a physiological pH, ensuring their suitability for a potential topical application on the skin. Alcohologels based on PA had an acidic pH, regardless of the formulation method. The alcohologels containing PA and modified starches showed a pH close to physiological skin surface pH (4.1–5.8) [60]. Z. Wang et al. [27] prepared a polyacrylate hydrogel containing CT in niosomes at pH 6.7, which was determined to be suitable for topical applications. The alcohologels based on MC, HEC and PAPC exhibited alkaline pH (Table 1). Acrylic acid polymers were used as a basement to ensure an acidic environment [61]. Acidic potential is imparted to these polymers by carboxyl groups, which dissociate at low pH to form negatively charged anions [62].

### 3.2. CT Release Evaluation

A controlled release study was conducted to elucidate the mechanism of CT release from the alcohologels, with experiments performed under various conditions. Most drugs are distributed in the body according to first-order kinetic models. This model is particularly appropriate because the biological half-life of the drug in the body is independent of the administered dose. Moreover, a statistical Weibull model provides the opportunity to compare the release mechanisms based on the shape parameter β.

The release of CT into phosphate buffer solution (PBS) demonstrated a low efficiency of approximately 3%, despite the addition of PEG 400 and the twofold addition of ethanol to the alcohologel, which was intended to enhance the solubility of CT in the acceptor solution. Consequently, we opted to utilize methanol as the acceptor solvent, due to its effectiveness as a solvent for CT. Regardless, the study of the release of CT into inorganic (PBS) and organic (methanol) media further demonstrated the dependence of the rate and efficiency of release on the type of crosslinker in the PA. The fastest (after 1.5 h) and greatest (approximately 23%) release of CT was into methanol from PA crosslinked with NaOH solution (N-PA) (Figure 1b). Both releases into PBS and methanol had similar stages of increase and decrease. The release into PBS followed a similar pattern irrespective of the crosslinker (Figure 1a). In the case of the CT release into methanol, the release from N-PA was faster and CT decomposition was also faster than from PA crosslinked with AMPD solution (A-PA). Release of CT from A-PA was less efficient (up to 13%), but the decomposition process was less turbulent than from N-PA. These observations were confirmed by the values of the rate constants of the release and decomposition kinetics (Table 3). We conducted a kinetic analysis of CT release into both PBS and methanol, fitting the data to zero-order, first-order kinetic models, as well as the Weibull statistical model. The fitting was based on the highest coefficient of determination (r^2^) values. No suitable fit to the zero-order model was found for any of the examined alcohologels. Given the nature of the increase and subsequent decrease in the amount of released CT, we divided the first-order model into two stages: the release process and the decomposition process. The rate constants (k) for both stages were analyzed separately, using a method based on the graphical calculation of the tangent of the slope of the line. A better fit to the first-order model was observed for CT released into PBS. The kinetics of the above processes were fitted to a non-linear Weibull model. Shape parameter (β) was calculated. For the process of release into PBS, β values were 0.9393 and 0.9669 for A-PA(i) and N-PA(i), respectively, while the β value for A-PA was 0.4701 and for N-PA, it was 4.2830. However, the very low values of the determination coefficient (r^2^) for the release processes of CT to methanol prevent these processes from being fitted to the Weibull model. The β parameter of the Weibull model can approximate information on the drug release process. A value of β ≤ 0.75 indicates Fick diffusion, and a β between 0.75 and 1.0 points to Fick diffusion combined with case II transport, while super case II drug transport has been reported for β values greater than 1.0 [63]. The release of CT into PBS was found to adhere to Fick’s first law, where the rate of change is proportional to the concentration gradient. However, this process also involves additional mechanisms, such as dissolution and interaction with the surrounding environment. In contrast, the process of CT release exhibited a more complex behavior when released into methanol. The release stage of CT from the alcohologel containing AMPD was more accurately represented by a first-order model, as was the decomposition stage of CT when released from the alcohologel containing NaOH. The release of CT showed a non-standard profile similar to drug levels in the biological models where elimination of the drug occurs, e.g., through its metabolism [64]. In the case of an in vitrum study, a CT breakdown reaction may be sought.

### 3.3. Determination of CT Stability

As previously mentioned, hydrogels are known to enhance the delivery and stability of compounds such as cryptotanshinone (CT) for topical applications. The addition of various conditions may influence both the stability and subsequent activity of CT in the studied alcohologels. The influence of alcohologel formulation parameters, such as the type of alkaline reagents, ultrasound at elevated temperature, and the addition of native and modified starch, on CT stability after 2 months was observed. The CT content measured after 60 days in the formulations stored at 8 °C was the highest for NaOH-crosslinked PA with SM2.5 starch (N-PA-SM2.5—approx. 70%) and the lowest for NaOH-crosslinked PA at 60 °C in the presence of ultrasound (N-PA(*)—0%) (Figure 2). Generally, higher CT contents were noted in NaOH-crosslinked PA, which was also confirmed by the results of the CT release (Figure 1b). In the case of N-PA(*), several factors that could influence the degradation of CT were considered. A temperature of 60 °C did not significantly affect the stability of CT, as confirmed by Z. Wang’s research on the stability of niosome systems containing CT in hydrogel [27]. Similarly, the ultrasound used by this researcher to prepare the niosomes with CT did not cause degradation; instead, it enhanced the stability of the system. Moreover, the temperature of 60 °C does not appear to be detrimental to CT, as its endothermic peak occurs at 193.6 °C. Additionally, CT was dried at 60 °C for the electrospray ionization mass spectrometry (ESI-MS) process. Similarly, ultrasonication at 40 kHz for 10 min at 60 °C did not seem critical, according to our previous study [4]. The key difference in this study was the concentration of NaOH used. In earlier tests, we evaluated the solubility of CT in a maximum of 4% NaOH solution, whereas in this study, CT was treated directly with an 18% NaOH solution. High concentrations of NaOH can cause the hydrolysis of compounds, potentially leading to their degradation. The use of a 10% NaOH solution (in combination with SnCl_2_) resulted in the alkaline hydrolysis of an aldehyde, leading to the synthesis of Tanshinone I by X. Huang and colleagues [65].

### 3.4. ATR-FTIR Analysis

**Figure 9:**
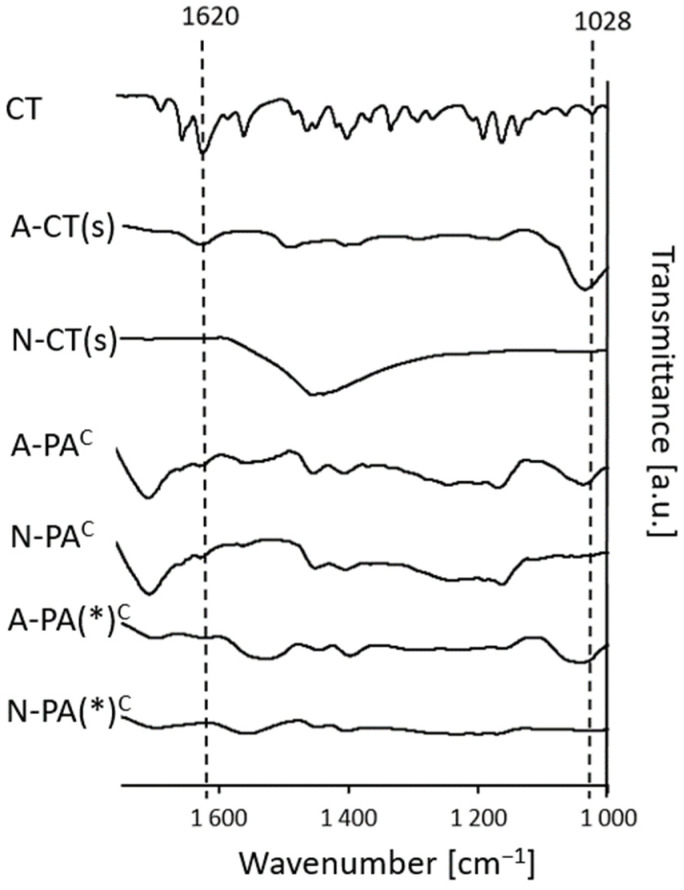
The FTIR spectra exhibited interactions between CT and. The asterisk (*) indicates alcohologels preparation method with higher concentrations of alkalizers and ultrasonication at 60 °C.

The ATR-FTIR study was evaluated to exhibit possible interactions between cryptotanshinone (CT) and other alcohologel components. The objective was to illustrate the interactions that could influence the release mechanism and the amount of cryptotanshinone (CT) released from alcohologels. The ATR-FTIR results showed the formation of amide bonds both in suspensions and in cryo-alcohologels containing CT and AMPD, at ca. 1520 and 1580 cm^−1^, respectively (Figure 3), indicating interactions between carboxyl and amine groups. Moreover, a significantly decreased absorption of hydroxide groups at around 600 cm^−1^ in the alcohologels containing CT, PA and AMPD, compared to pure and physical mixture of these components, may identify interactions between PA and AMPD. The interaction with the NaOH solution and PA might be electrostatic, as no additional peak was observed, indicating a covalent interaction between them. However, the peaks of antisymmetric stretching of the COO¯ at about 1550 cm^−1^ [52] and a broad peak at about 3370 cm^−1^ of O-H [53] were observed, likely attributed to the interactions between them. The formulations with PA had peaks at around 1700 cm^−1^, corresponding to stretching C=O vibration [50]. Kirwan et al. [52] observed the occurrence of symmetrical and asymmetrical vibrations in the ionized form of the carboxyl group of succinic acid at 1552 and 1396 cm^−1^ at pH 11.5, respectively, and for polyacrylate at 1562 and 1408 cm^−1^ at pH 13. At pH 6.5, only the unchanging values of the stretching vibration of the C=O group at 1650 cm^−1^ were observed. Polyacrylic acids changed their conformation depending on the change in pH. From pH 3.0, their carboxyl groups became ionized and were fully ionized at pH 9.0. As the pH increased above 7.0, the concentration of sodium ions also increased, which shielded the charge of the ionized carboxyl groups, counteracting their repulsion and formation into a coiled hydrogel form [66]. The spectra of cryo-alcohologels prepared by the two methods, including ultrasound and high temperature and without them, did not show significant differences. Demonstrating the interaction between CT and suspension or hydrogel components requires focusing on C-O absorption at 1028 cm^−1^ [67] occurring in the dihydrofuran ring, as well as on C=O absorption at 1620 cm^−1^ [37]. The spectra of the formulations containing NaOH show the absence of the C-O absorption band at 1028 cm^−1^, indicating an interaction between NaOH and cryptotanshinone (CT). The presence of the C=O absorption at 1620 cm^−1^ in the suspension of CT with NaOH further supports this interaction. However, in the case of alcohologels, the observed C=O absorption is likely attributed to the polymeric carboxyl groups. For alcohologels prepared at higher temperatures and with increased alkalizer concentrations, a significant decrease in the C=O absorption is observed, particularly with NaOH (Figure 9).

### 3.5. Powder X-Ray Diffraction (PXRD) Analysis

PXRD (powder X-ray diffraction) is a widely used technique for studying the crystalline structure of solid materials. Our aim was to identify the presence and determine the extent of CT in the investigated formulations. Additionally, by comparing the diffraction intensities of the polymers and alkalizing agents, we were able to assess the occurrence of interactions between them. The diffractogram of N-PA^c^ showed a reflection peak at 8.30°, while A-PA^c^ and A-PA(*)^c^ showed the shoulders at 8.34° and 8.34°, respectively, which correspond with diffraction peak of CT at 8.34° (Figure 4). Moreover, these formulations varied in intensity. The crystallinities were 2.9, 0.5 and 0.4% for N-PA^c^, A-PA^c^ and A-PA(*)^c^, respectively. The broad amorphous reflection of PA at 2θ value of about 18° was mostly identified in the formulations of PA containing AMPD. It was less visible in the formulations with NaOH, indicating a strong interaction between PA and AMPD. These results correspond with the S.-A. Chen and H.-T. Lee [68] observations on the association of PA with polyaniline.

### 3.6. Differential Scanning Calorimetry (DSC)

Differential scanning calorimetry(DSC) was used as a characterization technique to study the thermal properties and stability of the alcohologels. The DSC thermograms from the first and second heating runs exhibited notable differences. The first heating thermograms indicated volatinization of water contained within the polymeric matrix of amorphous PA at approximately 64 °C represented by a broad peak (Figure 5a). The peaks observed at higher temperatures in the formulations of PA with NaOH suggested an increase in stability [69]. The thermograms obtained after cooling determined the glass transition temperature (T_g_) of PA at 147.8 °C. S. Khanlari and M.A. Dubé [58] demonstrated the dependence of T_g_ of polyacrylic acid on the pH during polymerization, ranging from 60 to 150 °C at pH values of 4 to 7, respectively. D. de la Garza et al. [70] analyzed DSC thermograms of polyacrylic acid depending on the different scanning rates ranging from 1 to 50 °C min^−1^, reporting a T_g_ of 135.7 °C for PA tested at a rate of 10 °C min^−1^ from 40 to 225 °C. In our study, DSC analyses were conducted at a rate of 5 K min^−1^. The DSC scans of the crystalline compouns including CT, AMP and NaOH revealed sharp peaks during the first heating cycle, indicative of melting temperature (Figure 5a). AMPD displayed a transition temperature ranging from 80 to 110 °C, transitioning from a plastic crystalline to liquid state [56,71]. In our analysis, this transition occurred between 84.4 and 112.4 °C. Notably, the thermograms of the formulations of PA with CT during both the first and second heating cycles showed no significant differences compared to the reference samples without CT (Figure 5a,b).

### 3.7. ESI-MS Evaluation

Electrospray ionization mass spectrometry (ESI-MS) analysis enabled the determination of the molecular mass and structure of CT and AMPD molecules based on the analysis of their ions formed during the ionization process. This allowed for both qualitative and quantitative determination of CT content in suspensions containing AMPD, as well as in suspensions with NaOH, both with 10% ethanol addition. Additionally, by comparing the ion intensity of CT on day 1 and after 30 days, we were able to assess the stability of CT in these formulations. ESI-MS studies also provided insights into the structural disorganization of the CT molecule in the presence of NaOH solution.

**Figure 10:**
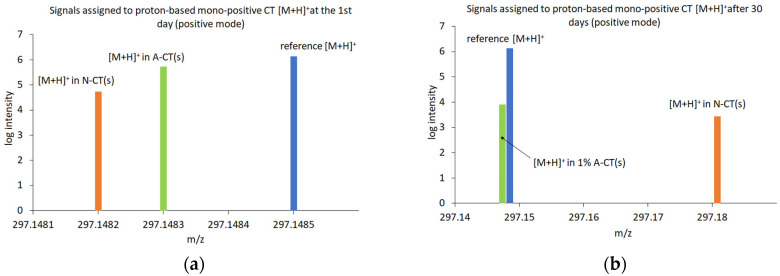
Comparison of the initial (**a**) and after 30 days (**b**) ESI-MS signals derived from CT at *m*/*z* 297.1485 ([M + H]^+^) dissolved in test solvents with a reference-calculated CT signal.

**Figure 11:**
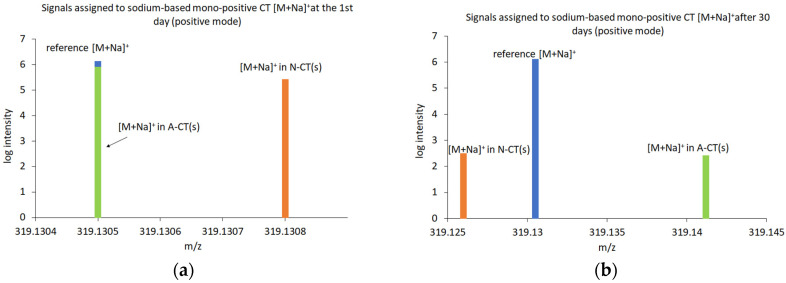
Comparison of the initial (**a**) and after 30 days (**b**) ESI-MS signals derived from CT at *m*/*z* 319.1305 ([M + Na]^+^) dissolved in test solvents with a reference-calculated CT signal.

The peaks of *m*/*z* values corresponding to the molecular weight of CT, T-V and AMPD, without proton [M − H]^−^, exhibited larger deviations and lower intensities compared to the peaks of the corresponding molecules containing proton [M − H]+ (Table 3). Figure 10 and Figure 11 reveal the signals assigned to CT with proton and sodium cation, respectively. The signal at *m*/*z* 297.1485 ([M + H]^+^) came from proton-based mono-positive CT (Figure 10) and at *m*/*z* 319.1305 ([M + Na]^+^) corresponded to a mono-positive sodium ion of CT (Figure 11).

An attempt to dissolve CT in 1% AMPD solution resulted in our obtaining two signals corresponding to the CT molecule, i.e.,: proton-based mono-positive ([M + H]^+^) and sodium-based mono-positive ([M + Na]^+^). The signals clearly indicated the presence of CT in the suspension (Figure 10a and Figure 11a). After a storage period of 30 days, the mono-positive hydrogen ion signal of CT was clearly identified, although with lower intensity compared to the measurements taken immediately after dissolving CT in 1% AMPD (Figure 10b). The second signal was ambiguous due to a significant deviation (Δ = −33.53) and exhibited low intensity (Figure 11b). A comparison of the signals and their intensities immediately after the dissolution of CT and after 30 days of storage indicates that CT was effectively dissolved in 1% AMPD and remained present in the suspension after 30 days, albeit in a reduced quantity.

As illustrated by the graphs, immediately after dissolving CT in 0.36% NaOH, high-intensity signals were obtained which closely aligned with the theoretically determined mass of the ionization product (Figure 10a and Figure 11a). However, in the same samples analyzed after 30 days, the signal intensity was significantly lower (Figure 11b), and a notable shift was observed on the *m*/*z* axis (Figure 10b). It can be assumed that upon mixing CT with 0.36% NaOH, a CT suspension was formed, during which decomposition processes occurred over approximately one month. Notably, the mono-positive sodium ion of CT proved to be more stable than the proton-based ion of CT.

**Figure 12:**
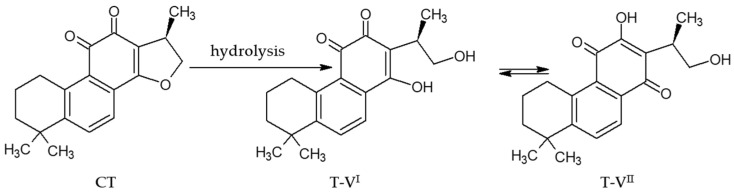
The structure of tanshinone V (T-V) after CT hydrolysis and its isomeric forms (I, II) [72].

Furthermore, the ESI-MS spectra of CT suspensions with 1% AMPD and 0.36% NaOH indicated the presence of the hydrolyzed form of CT–T-V and/or its sodium salt (Figure 7c–f, Table 3). The suspension of CT with 0.36% NaOH exhibited a signal for the sodium salt of T-V at *m*/*z* 359.123 ([M − H + 2Na]^+^), with an increase in intensity after 30 days of storage (Figure 7e,f, Table 3). The signal corresponding to the sodium-based mono-positive T-V at *m*/*z* 337.1410 ([M + Na]^+^) demonstrated instability in both the 1% AMPD and 0.36% NaOH solutions after approximately one month of storage (Figure 7d,f, Table 3). The most stable form for the CT with 1% AMPD solution was identified as the hydrogen-based mono-positive T-V at *m*/*z* 315.1591 ([M + H]^+^) (Figure 7c,d, Table 3). Sun et al. [72] investigated the metabolism of CT and other tanshinones in rat bile, establishing dehydrogenation, hydroxylation and dihydrofuran ring hydrolysis as the main metabolic pathway for CT (Figure 12). Wei et al. [17] studied the metabolism of CT and other tanshinones in Zebrafish, confirming similar metabolic processes. Dihydrofuran ring hydrolysis was also noted by An et al. [73]. The effects of selected amines on CT and tanshinone IIA (T-IIA) were investigated revealing that nucleophilic attack in ammonia solutions resulted in the opening of the dihydrofuran ring moiety of CT. Furthermore, the presence of CH_3_NH_2_ or RCH_3_NH_2_ solutions led to the formation of imidazole or oxazole ring in CT structure, respectively. In the case of T-IIA, there was no cleavage of the furan ring; instead, only the oxazole ring formation was observed.

### 3.8. SEM Images

SEM images were taken. The crystalline form of CT is shown on the first SEM micrograph (Figure 8A). A micrograph of the vacuum-dried CT suspension with AMPD and ethanol (Figure 8B) exhibits the greater degradation of the crystalline form of CT than in the case of interaction of CT with NaOH (Figure 8C). The photos present the silmilarity of cryo-alcohologels with PA, AMPD and CT structure to the stucture without CT (Figure 8E, 8F and 8D respectively). SEM images of CT-enhanced cryo-alcohologels showed irregularities. The application of elevated temperature and ultrasonication in case of AMPD addition was not noticed on the SEM micrographs (Figure 8E,F). The images of the cryo-alcohologels with NaOH present a perforated structure similar to a network (Figure 8G–I). This is in agreement with the SEM micrograph of PA mixed with attapulgite ((Mg,Al)_2_Si_4_O_10_(OH)·4(H_2_O)) [74], where the polymer structure has formed a network. The SEM image of the N-PA(*)^c^ cryo-alcohologel exhibits the most perforated structure (Figure 8I). It may confirm the destructive effect of simultaneous application of a temperature of 60 °C, an 18% NaOH solution and ultrasonication on the polymer structure and, at the same time, on the substances contained therein, or the strongest influence of one of them. As discussed above, regarding the stability factors of cryptotanshinone (CT), it appears that the high concentration of NaOH has the greatest impact on its stability. The use of high concentrations of NaOH to crosslink hydrogels takes place in order to achieve a highly porous hydrogel structure. Chavda and Patel [75] used 20% NaOH solution to obtain superporous gel base on polyacrylic acid and polyacrylamides.

## 4. Materials and Methods

Cryptotanshinone of 99.26% purity (CT) (Selleckchem, Poznań, Poland) in a powder form was used as a study material. Other reagents were as follows: methanol for HPLC ≥ 99.9% (CH_3_OH) (Chempur, Piekary Śląskie, Poland), sodium hydroxide (NaOH) (Stanlab Sp. J., Lublin, Poland) and 2-amino-2-methyl-1,3-propanediol (AMPD) (Sigma-Aldrich, Poznań, Poland). For alcohologel preparation, the following were used: polyacrylic acid—Carbopol^®^ 980NF (PA, Lubrizol, New Brunswick, NJ, USA), methyl cellulose (MC, Sigma-Aldrich, Poznań, Poland), hydroxyethylcellulose high-viscosity (HEC, Pol-Aura, Morąg, Poland), polyacrylic acid crosspolymer—Aristoflex^®^ Velvet (PACP, Crosspolymer 11 polyacrylate Clariant, Frankfurt, Germany), ethanol 96% (C_2_H_5_OH) (Stanlab Sp. J., Lublin, Poland), native potato starch (NS, PPZ, Niechlów, Poland), and modified starch hydrothermally hardened at 120 °C with 2.5% and 10% citric acid content (University of Environmental and Life Sciences, Wrocław, Poland). For HPLC, the following were used: formic acid (Sigma-Aldrich, Poznań, Poland) and acetonitrile (Pol-Aura, Morąg, Poland).

### 4.1. Alcohologels Preparation

Alcohologels were formulated using methylcellulose (MC), hydroxyethylcellulose (HEC), polyacrylic acid (PA, Carbopol^®^ 980 NF) and its derivative polyacrylic acid crosspolymer (PACP, Aristoflex^®^ Velvet) (Table 4). The ratios of hydrogel polymers to alkaline solvents were optimized for pH requirements, specifically 1.5:1.0 (*w*/*w*) for 2-amino-2-methyl-1-propanol (AMPD) and 1.6:0.36 (*w*/*w*) for sodium hydroxide (NaOH Additionally, CT was dissolved in 96% ethanol at concentrations of 0.140% and 0.016%, used in ratios of 2:10 and 1:10 (*w*/*w*) within the alcohologels for subsequent release studies in phosphate-buffered saline (PBS) and other experiments. Briefly, hydrogel polymers were combined with solvent solutions, mixed in an unquator and stored at 8 °C for 12 h. MC and HEC were prepared at 70 °C. Reference alcohologels devoid of CT were synthesized via analogous procedures. The chosen reference alcohologels without CT were prepared similarly. Formulations containing PA were developed under varying physical conditions. Specifically, the CT–ethanol solution was combined with alkaline solutions (2.3% AMPD or 18% NaOH) and subjected to ultrasonication at 40 kHz (Sonic-5, Polsonic, Warsaw, Poland) at 60 °C prior to integration for ten minutes with hydrogel polymers (denoted with “(*)” in Table 4). All alcohologels were stored at 8 °C throughout the examination period. Notably, the PA-containing formulations were augmented with native potato starch (SN) at 17.5 ± 1.0% (m/m) along with modified starches featuring 2.5% (SM2.5) and 10% (SM10) citric acid substitutions (Table 4).

### 4.2. Determination of pH

The pH values of the resulting alcohologels were measured at 22.0 ± 1.0 °C. The concentrations and proportions of the ingredients were chosen to maintain the range of pH for human skin. The pH values of the alcohologels were measured by a pH meter, CPC-511, with a pH accuracy of ± 0.01 (Elmetron Sp.j., Zabrze, Poland) and pH electrodes, ERH-11S (Hydromet Sp.c., Gliwice, Poland). Each alcohologel was measured four times. A statistical ANOVA test with the threshold for significance set at 0.05 (5%) was performed.

### 4.3. CT Release Evaluation

#### 4.3.1. CT Release into PBS

CT release was evaluated according to the pharmacopeial method in a paddle-over-disc apparatus (ERWEKA DT126 light, Langen, Germany). CT, of 0.14% concentration, appearing in alcohologels of PA with AMPD or NaOH with 20% ethanol (A-PA(i), N-PA(i), respectively) was released into 300 mL of the mixture of phosphate buffer pH 5.5 (PBS) with PEG 400 at a volume ratio of 3:2. Experimental conditions were set at 37.0 ± 0.5 °C and paddle speed 50 rpm. Samples of 0.5 mL each were taken and analyzed using high-performance liquid chromatography (HPLC) according to our previous method [4], and the chamber was refilled with the same amount of pure medium after collection. Samples were taken within 94 h, at intervals of no less than one hour. The mobile phase was composed of distilled water and 0.1% formic acid (phase A) and acetonitrile and 0.1% formic acid (phase B). The gradiented A/B was set, respectively, as 98:2–1:99 and 1:99–98:2 *v*/*v*% for 28 min. A wavelength of 280 nm, a flow rate of 0.4 mL/min and a column temperature of 40 °C were established. The HPLC system (Ultimate 3000 model, Thermo Fisher Scientific, Schwerte, Germany), which consisted of a pump (LPG-3400SD), an autosampler (WPS-3000TSL), a column thermostat (TCC-3000SD), a UV detector (UV, DAD-3000) and a column (Kinetex 2.6 μm C18 100Å, Phenomenex, Torrance, CA, USA), was applied. The solutions of 99.26% pure CT in methanol for HPLC (≥99.9%) were prepared to a standard curve.

#### 4.3.2. CT Release into Methanol

The CT release was evaluated by employing a pharmacopeial Franz chamber apparatus. The 0.016% CT was released from the alcohologels of PA with AMPD or NaOH with 10% ethanol (A-PA or N-PA, respectively) into methanol. The parameters of the process were 22.0 ± 0.5 °C, constant flow rate of 2 mL/min, 6-h release with sampling every 30 min. The CT release amount was obtained using HPLC, described in Section 4.3. in the previous paragraph.

The releases were analyzed on the basis of the following kinetic models:

Zero-order kinetics (1)

(1) $$Q_{t}=Q_{0}-K_{\left(0\right)}t$$

and the Weibull model (2)

(2) $$\frac{Q_{t}}{Q_{e}}=1-e^{\frac{{-t}^{\beta }}{\alpha }}$$

where *K*_(0)_—rate constant of the zero-order kinetic model; *t*—time; *Q*_0_—initial percentage of the released drug; *Q_t_*—percentage of the released drug after time; *Q_e_*—percentage of the released drug in maximum state; α and β are the scale and shape parameters in the Weibull model. Non-linear estimation of the Levenberg–Marquardt method by Statistica 13.1 software was established with a confidence level of 95% (α = 0.05).

The rate constants for the release (k_r_) and decomposition (k_d_) processes were determined using a graphical method by subtracting the experimentally found CT concentrations from the values found on the extension of the descending part of the curve. The rate constants were calculated according to the Equation (3)

(3) $$k_{i}=\frac{lnC_{1}-lnC_{2}}{t_{2}-t_{1}}$$

### 4.4. Determination of CT Stability

The content of CT in the selected alcohologels was tested using the HPLC method, described above in CT release evaluation section (see Section 4.3). The influence of the composition of the alkaline substances, addition of native and modified starch and the use of elevated temperatures and ultrasound during the alcohologel preparation on CT levels was investigated in comparison with the CT content of the alcohologels alone prepared at 22.0 ± 0.5 °C. The obtained alcohologels were dissolved in 5 g of 96% ethanol using an orbital shaker for 30 min at 300 rpm and filtered through the 0.22 µm pores. The HPLC measurements were performed after 60 days of alcohologel storage under refrigerated conditions. Measurement of humidity loss during storage was carried out and included in the measurement results.

### 4.5. Preparation of the Samples for Spectral and Thermal Studies and Microscope Visualization

Freeze-drying of alcohologels was performed in order to receive ATR-FTIR, PXRD, DSC and SEM images. The alcohologel samples were dried in a double cycle of 24 h; 22 h of freezing at the temperature of −34 °C and vacuum 0.25 mbar and drying for 2 h, at the temperature of −47 °C and vacuum 0.055 mbar using Freeze Dryer (ALPHA 2–4 LDplus, Christ, Osterode am Harz, Germany).

### 4.6. ATR-FTIR Analysis

The FTIR study was carried out by an infrared spectrophotometer with Fourier transformation (FTIR) and attenuated total reflectance (ATR) appetizer (Nicolet 380 FTIR, Thermo Scientific, Waltham, MA, USA) with OMNIC software version 5.0. The alcohologel samples (A-PA(0), A-PA, A-PA(*),N-PA(0), N-PA and N-PA(*)) were freeze-dried and grated to a powder in an agate mortar. The suspension (A-CT(s) and N-CT(s)) were vacuum-dried and grated. The physical mixtures (A-PA(mix) and N-PA(mix)) and pure ingredients (CT, AMPD, NaOH and PA) were similarly grated. The spectra of powders were recorded over a wavelenght of 400 cm^−1^ to 4000 cm^−1^ at 32 scans per sample and a resolution of 4 cm^−1^. They were analyzed using a dedicated computer program (OMNIC Spectra 2.0).

### 4.7. Powder X-Ray Diffraction (PXRD) Analysis

The powder PXRD data were recorded on a Bruker D2 PHASER diffractometer (Bruker AXS, Karlsruhe, Germany) with a Lynxeye detector using Cu Kα radiation (1.5418 Å). Cryo samples of alcohologels (A-PA(0), A-PA, A-PA(*),N-PA(0), N-PA and N-PA(*)) grated to a powder in an agate mortar, the physical mixtures of the corresponding ingredients (A-PA(mix) and N-PA(mix)) and pure substances (CT, AMPD, NaOH and PA) were measured at 295 K with a 0.5 mm slit and 1.0 mm shutter. Diffractograms were obtained between 5° and 55° (2θ) (step size of 0.02° (2θ) and 0.5 s/step). The X-ray generator operated at 30 kV and 10 mA. The PXRD patterns were processed using the software Diffrac.Eva V 3.2. (Bruker AXS). The proportion of crystallinity and amorphousness for the tested samples was determined using the amorphous subtraction method [76,77].

### 4.8. Differential Scanning Calorimetry (DSC)

The differential scanning calorimeter DSC 214 (Polyma, Netzsch, Selb, Germany) equipped with an intracooler IC70 (Netzsch, Selb, Germany) was used. For determination of the plasticizing effect of absorbed water, the heating–cooling–heating cycle was as follows: −10 °C/+300 °C/ 0 °C/−10 °C/+300 °C at a rate of 5 K/min in a nitrogen atmosphere. Cryo samples of 3–5 mg of alcohologels (A-PA(0), A-PA, A-PA(*), N-PA(0), N-PA and N-PA(*)) grated to a powder in an agate mortar, their ingredients (CT, AMPD, NaOH and PA) and the physical mixtures of the corresponding ingredients (A-PA(mix) and N-PA(mix)) were analyzed in aluminium pans. The crucibles were closed with a lid perforated with a needle. The obtained DSC curves were analyzed by an applicable Proteus^®^ software (Netzsch, Selb, Germany, Proteus for Thermal Analyzers—NETZSCH Analyzing & Testing, https://analyzing-testing.netzsch.com/en-AU/products/software/proteus, accessed on 2 December 2024)

### 4.9. Electrospray Ionization Mass Spectrometry (ESI-MS) Analysis

The ESI-MS analysis was performed on CT suspensions with alkaline components on the first day of manufacture and after 30 days of storage at 22 ± 2 °C without light, on a compact™ ESI-TOF mass spectrometer (Bruker Daltonics, Bremen, Germany) equipped with a standard ESI source. The instruments were operated in positive and negative ionization mode. A-CT(s) and N-CT(s) were prepared in ultrapure water and filtered through 0.22 µm pore-diameter filters and then dried at a temperature of 60 °C and dissolved in methanol. The methanolic and ethanolic solutions of CT were proposed as standards. *m*/*z* and intensity values as well as the isotope distribution of the peaks of the components of the solutions were compared to the exemplary spectra of the solvents and CT on the basis of their molecular weight. The obtained data were processed with the Compass DataAnalysis 4.2 software package (Bruker, Bremen, Germany).

### 4.10. Scanning Electron Microscope (SEM) Images

Microscope images revealed the structure of alcohologels with various solvents and preparation methods. The samples for SEM images were plated with gold, and the microphotographs were taken using scanning electron microscope (Quanta 650 FEG, Thermofisher Scientific) at 100, 12,000 and 15,000× magnification.

## 5. Conclusions

The release rate of CT into the methanol acceptor compartment was more emphasized in alcohologels with NaOH compared to alcohologels with AMPD, which was confirmed by observation of intensity of CT peaks in PXRD as well as through the FTIR analysis of desiccated alcohologels. The presence of the 0.36% NaOH addition promoted CT stability in assessed alcohologels evaluated in HPLC studies. SEM visualization of CT samples alkalized by AMPD or NaOH and vacuum-dried confirmed a more destructive influence on CT in the samples containing 1% AMPD compared to 0.36% NaOH. CT in the samples prepared by the method with the use of a high concentration of NaOH, i.e., 18%, was more destructive compared to the CT in the samples prepared with the use of a relatively high concentration, 2,3% AMPD, which was visible in the HPLC stability studies, as well as in the PXRD measurements of desiccated alcohologels. This result range indicates that the destructive effect of a specific alkalizing component, added in high concentrations, to a preparation with CT depends both on the strength of the electrolyte as well as on the concentration range. In complex systems, it should be predictable with detailed measurements. The modified starch, mostly SM2.5, in the presence of 0.36% NaOH, increased the CT stability in the alcohologel systems according to an HPLC stability assay. The SEM images of polymeric alcohologels with CT, taken after freeze-drying, confirmed the destructive effect of high initial concentrations of NaOH, sonification and increased temperature on the structure of evaluated systems with CT. The increased porosity in the desiccated samples of the polymer system was observed when a high concentration of NaOH was used during its preparation. According to the ESI-MS assay, in methanol and NaOH, the CT most likely transformed into tanshinone V sodium.

## Figures and Tables

**Table 1:** The pH values and dynamic viscosity of alcohologels with or without CT and starches dissolved in the ethanol solution doped with 0.36% NaOH or 1.0% AMPD. Meaning of abbreviations and symbols: A—2-amino-2-methyl-1,3-propanediol (AMPD), N—sodium hydroxide (NaOH), PA—polyacrylic acid, SN—native starch, SM2.5—modified starch with 2.5% citric acid, SM10—modified starch with 10% citric acid, MC—methylcellulose, HEC—hydroxyethylcellulose, PACP—polyacrylic acid crosspolymer, (0)—alcohologels without CT, (*)—alcohologels prepared with higher concentrations of alkalizers and ultrasonication at 60 °C.

Alcohologel Samples	pH Values	
	Mean	SD
A-PA(0)	6.46	0.02
A-PA	6.11	0.01
A-PA(*)	5.99	0.11
A-PA-SN	6.07	0.03
A-PA-SM2.5	5.64	0.02
A-PA-SM10	5.23	0.01
A-MC	10.40	0.02
A-HEC	10.14	0.02
A-PACP	9.93	0.03
N-PA(0)	6.81	0.02
N-PA	5.94	0.01
N-PA(*)	5.79	0.00
N-PA-SN	6.07	0.03
N-PA-SM2.5	5.32	0.02
N-PA-SM10	5.00	0.03
N-MC	12.93	0.02
N-HEC	12.90	0.08
N-PACP	13.39	0.07

**Table 2:** Kinetic model parameters of CT release into PBS and methanol; K_0_—zero rate constant, K_r_—release rate constant, K_d_—decomposition rate constant, β—shape constant, α—scale constant, r^2^—determination coefficient; *n* = 3.

Release Medium	Sample	Parameters of Release Kinetics													
		The Zero-Order Kinetics			The First-Order Kinetics, when Two Parallel Processes Were Assumed: Drug Release into the Acceptor Compartment and Drug Decomposition						Weibull Model				
					Release Kinetics Parameters			Decomposition Kinetics Parameters							
		K_0_ [%·h^−1^]	SD	r^2^	K_r_ [h^−1^]	SD	r^2^	K_d_ [h^−1^]	SD	r^2^	β [-]	SD	α [-]	SD	r^2^
PBS	A-PA(i)	0.0185	0.0030	0.5418	0.0644	0.0044	0.9978	0.0078	0.0018	0.9999	0.9393	0.1893	9.0914	2.5880	0.8688
	N-PA(i)	0.0144	0.0010	0.5008	0.0653	0.0166	0.9913	0.0076	0.0017	0.9999	0.9669	0.0511	8.8245	1.3219	0.9026
MET	A-PA	0.2598	0.2837	0.7887	1.9741	1.2063	0.9324	0.0444	0.0140	0.5365	0.4701	0.3557	0.7699	0.0528	0.3699
	N-PA	−0.7465	0.1069	0.0957	3.3992	0.3304	0.8610	0.2285	0.0064	0.9918	4.2830	0.3314	0.1198	0.0423	0

**Table 3:** Identification of compounds produced exclusively by the process—in ESI-MS analysis, which has isotope representation, deviation < 40 ppm. Shortcuts: calc.—calculated, emp.—empiric, diff.—difference, n.ap.—not applicable.

Ion Descriptions				Initially			After 30 Days		
Formulation	Components	Isotopic Form	Calc. [*m*/*z*]	Emp. [*m*/*z*]	Diff. [ppm]	Intensity	Emp. [*m*/*z*]	Diff. [ppm]	Intensity
MeOH-CT(sol)	CT	[M + H]^+^	297.1485	297.1472	4.37	110,000	n.ap.	-	-
	CT	[M + Na]^+^	319.1305	319.1288	5.33	20,000	n.ap.	-	-
ETOH-CT(sol)	CT	[M + H]^+^	297.1485	297.1468	5.72	20,000	n.ap.	-	-
	CT	[M + Na]^+^	319.1305	319.129	4.70	20,000	n.ap.	-	-
A-CT(s)	AMPD	[M *−* H]^−^	104.0706	104.0739	−31.71	200,000	n.ap.	-	-
	T-V	[M *−* H]^−^	313.1434	313.1507	−23.31	400,000	n.ap.	-	-
	CT	[M *−* H]^−^	295.1329	295.1408	−26.77	500	n.ap.	-	-
	AMPD	[M + H]^+^	106.0863	106.0862	0.94	2,500,000	106.0855	7.54	5,500,000
	AMPD	[M + Na]^+^	128.0682	128.068	1.56	4,000,000	128.0674	6.25	100,000
	CT	[M + H]^+^	297.1485	297.1483	0.67	400,000	297.1474	3.70	6000
	CT	[M + Na]^+^	319.1305	319.1305	0.00	600,000	319.1412	−33.53	200
	T-V	[M + H]^+^	315.1591	315.1584	2.22	8000	315.159	0.32	7000
	T-V	[M + Na]^+^	337.141	337.1404	1.78	50,000	337.1405	1.48	2000
N-CT(s)	T-V	[M *−* H]^−^	313.1434	313.1458	−7.66	150,000	n.ap.	-	-
	CT	[M + H]^+^	297.1485	297.1482	1.01	100,000	297.1808	−108.70	2000
	CT	[M + Na]^+^	319.1305	319.1308	−0.94	200,000	319.126	14.10	240
	T-V	[M + H]^+^	315.1591	315.1593	−0.63	200,000	-	-	-
	T-V	[M + Na]^+^	337.141	337.1416	−1.78	600,000	337.1436	−7.71	2000
	T-V sodium	[M *−* H + 2Na]^+^	359.123	359.1231	−0.28	70,000	359.1219	3.06	400,000

**Table 4:** The composition of the alcohologels and mixtures.

	Ingredient	CT [g]	ETOH [g]	NaOH [g]	AMPD [g]	H_2_O [g]	PA [g]	MC [g]	HEC [g]	PACP [g]	SN [g]	SM2.5 [g]	SM10 [g]
Sample													
A-PA(0)		-	10.0	-	1.0	87.5	1.5	-	-	-	-	-	-
A-PA		0.016	10.0	-	1.0	87.5	1.5	-	-	-	-	-	-
A-PA(*)		0.016	10.0	-	1.0	87.5	1.5	-	-	-	-	-	-
A-PA(i)		0.140	20.0	-	1.0	77.4	1.5	-	-	-	-	-	-
A-PA-SN		0.015	10.0	-	1.0	69.0	1.5	-	-	-	18.5	-	-
A-PA-SM2.5		0.015	10.0	-	1.0	69.3	1.5	-	-	-	-	18.2	-
A-PA-SM10		0.015	10.0	-	1.0	69.0	1.5	-	-	-	-	-	18.5
A-MC		0.016	10.0	-	1.0	88.0	-	1.0	-	-	-	-	-
A-HC		0.016	10.0	-	1.0	88.0	-	-	1.0	-	-	-	-
A-PACP		0.016	10.0	-	1.0	87.5	-	-	-	1.5	-	-	-
A-PA(mix)		0.001	-	-	0.002	-	0.002	-	-	-	-	-	-
A-CT(s)		0.0016	1.0		0.09	8.9	-						
N-PA(0)		-	10.0	0.36	-	88.0	1.6	-	-	-	-	-	-
N-PA		0.016	10.0	0.36	-	88.0	1.6	-	-	-	-	-	-
N-PA(*)		0.016	10.0	0.36	-	88.0	1.6	-	-	-	-	-	-
N-PA(i)		0.140	20.0	0.36	-	77.9	1.6	-	-	-	-	-	-
N-PA-SN		0.014	10.0	0.36	-	70.2	1.6	-	-	-	17.8	-	-
N-PA-SM2.5		0.015	10.0	0.36	-	70.1	1.6	-	-	-	-	17.9	-
N-PA-SM10		0.015	10.0	0.36	-	71.3	1.6	-	-	-	-	-	16.7
N-MC		0.016	10.0	0.36	-	88.6	-	1.0	-	-	-	-	-
N-HC		0.016	10.0	0.36	-	88.6	-	-	1.0	-	-	-	-
N-PACP		0.016	10.0	0.36	-	88.1	-	-	-	1.5	-	-	-
N-PA(mix)		0.0006	-	0.002	-	-	0.0015	-	-	-	-	-	-
N-CT(s)		0.0016	1.0	0.036		9.06	-						

* preparation method with higher concentrations of alkalizers and ultrasonication at 60 °C.

## Data Availability

Data are contained in the article.
